# Development and validation of the Evidence Based Medicine Questionnaire (EBMQ) to assess doctors’ knowledge, practice and barriers regarding the implementation of evidence-based medicine in primary care

**DOI:** 10.1186/s12875-018-0779-5

**Published:** 2018-06-23

**Authors:** Ranita Hisham, Chirk Jenn Ng, Su May Liew, Pauline Siew Mei Lai, Yook Chin Chia, Ee Ming Khoo, Nik Sherina Hanafi, Sajaratulnisah Othman, Ping Yein Lee, Khatijah Lim Abdullah, Karuthan Chinna

**Affiliations:** 1 Department of Primary Care Medicine, Faculty of Medicine, University of Malaya, Kuala Lumpur, Malaysia; 2 Department of Family Medicine, Faculty of Medicine and Health Sciences, Universiti Putra Malaysia, UPM Serdang, Selangor, Malaysia; 3 Department of Nursing Sciences, Faculty of Medicine, University of Malaya, Kuala Lumpur, Malaysia; 4 Department of Social and Preventive Medicine, Julius Centre University of Malaya, University of Malaya, Kuala Lumpur, Malaysia; 5 Department of Medical Sciences School of Healthcare and Medical Sciences Sunway University, Selangor, Malaysia

**Keywords:** Evidence-based medicine, Primary care physicians, Attitudes, Questionnaire

## Abstract

**Background:**

Evidence-Based Medicine (EBM) integrates best available evidence from literature and patients’ values, which then informs clinical decision making. However, there is a lack of validated instruments to assess the knowledge, practice and barriers of primary care physicians in the implementation of EBM. This study aimed to develop and validate an Evidence-Based Medicine Questionnaire (EBMQ) in Malaysia.

**Methods:**

The EBMQ was developed based on a qualitative study, literature review and an expert panel. Face and content validity was verified by the expert panel and piloted among 10 participants. Primary care physicians with or without EBM training who could understand English were recruited from December 2015 to January 2016. The EBMQ was administered at baseline and two weeks later. A higher score indicates better knowledge, better practice of EBM and less barriers towards the implementation of EBM. We hypothesized that the EBMQ would have three domains: knowledge, practice and barriers.

**Results:**

The final version of the EBMQ consists of 80 items: 62 items were measured on a nominal scale, 22 items were measured on a 5 point Likert-scale. Flesch reading ease was 61.2. A total of 343 participants were approached; of whom 320 agreed to participate (response rate = 93.2%). Factor analysis revealed that the EBMQ had eight domains after 13 items were removed: “EBM websites”, “evidence-based journals”, “types of studies”, “terms related to EBM”, “practice”, “access”, “patient preferences” and “support”. Cronbach alpha for the overall EBMQ was 0.909, whilst the Cronbach alpha for the individual domain ranged from 0.657–0.940. The EBMQ was able to discriminate between doctors with and without EBM training for 24 out of 42 items. At test-retest, kappa values ranged from 0.155 to 0.620.

**Conclusions:**

The EBMQ was found to be a valid and reliable instrument to assess the knowledge, practice and barriers towards the implementation of EBM among primary care physicians in Malaysia.

**Electronic supplementary material:**

The online version of this article (10.1186/s12875-018-0779-5) contains supplementary material, which is available to authorized users.

## Background

Evidence-based medicine (EBM) is defined as the integration of best available evidence in a conscientious, explicit and judicious manner from literature and patients’ values which then informs clinical decision making [1]. Practicing EBM in clinical practice helps doctors make a proper diagnosis and selects the best treatment available to treat or manage a disease [2]. The use of EBM in clinical setting is thought to provide the best standard of medical care at the lowest cost [3].

Evidence-based medicine has an increasing impact in primary care over recent years [4]. It involves patients in decision making and influences the development of guidelines and quality standards for clinical practice [4]. Primary care physicians are the first person of contact for patients [5]. They have high workload and at the same time they need to uphold the quality of healthcare [6]. Therefore, it is important for them to treat patients based on research evidence, clinical expertise and patient preferences [7]. However, integrating EBM into clinical practice in primary care is challenging as there are variations in team composition, organisational structures, culture and working practices [8].

A search from literature revealed that the international main barriers were lack of time, lack of resources, negative attitudes towards EBM and inadequate EBM skills [9]. A recent qualitative study conducted in 2014 found that the unique barriers in implementing EBM among primary care physicians in Malaysia were lack of awareness and attention toward patient values. Patient values forms a key element of EBM and they still preferred obtaining information from their peers and interestingly, they used WhatsApp—a smart phone messenger [10].

Therefore, we need an instrument to determine the knowledge, practice and barriers of the implementation of EBM among the primary care physicians. It is important to have an instrument to identify the gaps on a larger scale and improve the implementation of EBM in their clinical practice. A systematic review by Shaneyfelt et al. [11] reported that 104 instruments have been developed to evaluate the acquisition of skills by healthcare professionals to practice EBM. These instruments assessed one or more of the following domains on EBM: knowledge, attitude, search strategies, frequency of use of evidence sources, current applications, intended future use and confidence in practice. However, only eight instruments were validated: four instruments assessed the competency in EBM teaching and learning [12–16], whilst four assessed knowledge, attitude and skills [16–19]. However, no instrument has assessed the knowledge, practice and barriers in the implementation of EBM. Therefore, this study aimed to develop and validate the English version of the Evidence-Based Medicine Questionnaire (EBMQ), which was designed to assess knowledge, practice and barriers of primary care physicians regarding the implementation of EBM.

## Methods

### Development of the evidence-based medicine questionnaire

A literature search was conducted in PubMed; using keywords such as “Evidence-based medicine”, “general practioners”, “primary care physicians” and “survey/questionnaire” from this search, nine relevant studies were identified [12–16, 19, 20]. However, only one instrument [20] evaluated the attitude and needs of primary care physicians. Twenty four items from this questionnaire and findings from two previous qualitative studies in rural and urban primary care settings in Malaysia [10, 21] were used to develop the EBMQ (version 1). The EBMQ was developed in English, as English is used in the training of doctors in medical schools and also taught as a second language in all public schools in Malaysia.

Face and content validity of the EBMQ was verified by an expert panel which consisted of nine academicians (a nurse, a pharmacist and seven primary care physicians). Each item was reviewed, and the relevance and appropriateness of each item was discussed (version 2). A pilot test was then conducted on ten medical officers with a minimum of one year working experience wihout any postgraduate qualification. They were asked to evaluate verbally if any items were difficult to understand. Feedback received were that the font was too small and that there was no option for “place of work” for those working in a University hospital. Changes were made based on these comments to produce version 3, which was then pilot tested in another two participants. No difficulties were encountered. Hence, version 3 was used as the final version.

### The evidence based medicine questionnaire (EBMQ)

The EBMQ consists of 84 items and 6 sections as shown in Table 1. Only 55 items (33 items in the “knowledge” domain, 9 items in the “practice” domain and 13 items in the “barriers” domain) were measured on a Likert-scale, and could be validated. The final version of the EBMQ is added in Additional file 1. A higher score indicates better knowledge and better practice of EBM and less barriers in practicing EBM.

**Table 1:** The initial version of the Evidence-Based Medicine Questionnaire (version 3)

Section	Description	No. of items	Domain	Type of data	Response options	Response combined for analysis
A	Demographic profile	6	NA	Nominal scale		
B	Frequencies in looking for medical information	20	NA	Nominal scale		
C	Knowledge regarding evidence-based medicine	17	Knowledge regarding information sources	4-point Likert scale^a^	1 = Unaware 2 = Aware but not used in clinical decision making 3 = Have read it but not used in clinical decision making 4 = Read and used in clinical decision making	
		16	Knowledge regarding terms related to EBM	5-point Likert scale^a^	1 = Never heard this term before 2 = Heard of this term but do not understand what this term but would like to 3 = Do not understand this term but would like to 4 = Have some understanding of this term 5 = Understand this term well and able to explain what it means to others	1 = Never heard and do not understand 2 = Do not understand but would like to 3 = Understand
D	Practice of evidence-based medicine	9	Practice	5-point Likert scale^a^	1 = Strongly disagree 2 = Disagree 3 = Neither agree nor disagree 4 = Agree 5 = Strongly agree	1 = Disagree 2 = Neutral 3 = Agree
E	Barriers in practicing evidence-based medicine	13	Barriers	5-point Likert scale^a^	1 = Strongly disagree 2 = Disagree 3 = Neither agree nor disagree 4 = Agree 5 = Strongly agree	1 = Disagree 2 = Neutral 3 = Agree
F	Needs for evidence-based medicine	3	Needs	Nominal scale		
	Total	80				

*NA* Not applicable

^a^Only items in these domain were tested for construct validity

Participants took 15 to 20 min to complete the EBMQ. We hypothesized that the EBMQ would have 3 domains: knowledge, practice and barriers.

### Validation of the evidence-based medicine questionnaire

#### Participants

Primary care physicians with or without EBM training, who could understand English and who attended a Diploma in Family Medicine workshop, were recruited from December 2015 to January 2016.

#### Sample size

Sample size calculation was based on a participant to item ratio of 5:1 to perform factor analysis [22]. There are 55 items in the EBMQ. Hence, the minimum number of participants required was 55*5 = 275.

#### Procedure

Permission was obtained from the Academy of Family Physicians Malaysia to recruit participants who attended their workshops. For those who agreed, written informed consent was obtained. Participants were then asked to fill in the EBMQ at baseline. Two weeks later, the EBMQ was mailed to each participant, with a postage-paid return envelope. If a reply was not obtained within a week, participants were contacted via email and/or SMS, and reminded to send in their completed EBMQ form as soon as possible.

#### Data analysis

Data were analyzed using the Statistical Package for Social Sciences (SPSS) version 22 software (Il, Chicago, USA). Normality could not be assumed, hence non-parametric tests were used. Categorical variables were presented as percentage and frequencies, while continuous variables were presented as median and interquartile range (IQR).

### Validity

#### Flesch reading ease

The readability of the EBMQ was assessed using Flesch reading ease. This was calculated based on the average number of syllables per word and the average number of words per sentence [23]. An average document should have a score of 60–70 [23].

#### Exploratory factor analysis

Exploratory factor analysis (EFA) was used to test the underlying structures within the EBMQ. EFA is a type of factor analysis that is utilised to identify the number of latent variables that underlies an entire set of items [24]. EFA was performed to explore the factors appropriateness that can be grouped into specific factors and also to provide information about the validity of each item in each domain. It is important to ensure that the items in each domain of the EBMQ are connected to their basic factors.

Factor loadings were assessed using the Keiser-Meyer-Olkin (KMO) and Bartlett’s test of sphericity. The principal components variance with promax variation were used for data reduction purposes, and eigenvalues > 1 was selected to see the variances of the principal components. KMO value of > 0.6, individual factor loadings > 0.5, average variance extracted (AVE) > 0.5 and composite reliability (CR) > 0.7, indicate good structure within the domains [25, 26].

#### Discriminative validity

To assess discriminative validity, participants were divided into those with or without EBM training. We hypothesized that the knowledge and practice of participants with EBM training would have better knowledge, better practice and less barriers than those without EBM training. The Chi-square test was used to determine if there was any difference between the two groups. A *p*-value < 0.05 was considered as statistically significant.

### Reliability

#### Internal consistency

Internal consistency was performed to test the consistency of the results and estimates the reliability of the items in the EBMQ. The internal consistency of the EBMQ was assessed using Cronbach’s α coefficient. A Cronbach’s alpha value of 0.5–0.69 is acceptable, while values of 0.70–0.90 indicate a strong internal consistency [27]. Corrected item-total correlations should be > 0.2 for it to be considered acceptable [28]. If omitting an item increases the Cronbach’s α significantly, the item will be excluded.

#### Test-retest reliability

The test-retest was performed to measure the reliability and stability of the items in the EBMQ over a period of time. It is also important to administer the same test twice to measure the consistency of the answers by the participants. The intra-class correlation coefficient (ICC) was used to assess the total score at test-retest. A ICC agreement value of 0.7 was considered acceptable [29]. ICC values between 0.75 and 1.00 indicate high reliability, 0.60 and 0.74 indicate good reliability, 0.40–0.59 has fair reliability and those below 0.40 indicate low reliability [30].

## Results

A total of 343 primary care doctors were approached; of whom 320 agreed to participate (response rate = 93.2%). The majority of them were female (69.4%) with a median age of 32.2 years [IQR = 4.0]. Nearly all (97.2%) were medical officers, working in government health clinics (54.4%) and possessed no postgraduate qualifications after their basic medical degree (78.4%). All participants had heard about EBM, but only 222 (69.7%) had attended an EBM course (Table 2).

**Table 2:** Demographic characteristics of participants

	n (%)
Median age [IQR]	32.2 [4.0]
Female	222 (69.4)
Male	98 (30.6)
No. of participants with postgraduate qualifications	
None	251 (78.4)
Diploma	58 (18.1)
Masters	11 (3.4)
Current designation	
Medical Officer	311 (97.2)
Family Medicine Specialist	9 (2.8)
Current Work Place	
Government health clinics	174 (54.4)
Private clinic	81 (25.3)
Government hospital	42 (13.1)
Others^a^	13 (4.1)
Private hospital	5 (1.6)
University hospital	5 (1.6)
Have heard of the term “evidence-based medicine”	319 (99.7)
Have attended EBM courses	222 (69.7)
Have received formal trainings in literature search	156 (48.8)
Have received formal trainings in questions formulation	121 (37.8)
Have received formal trainings in critical appraisal	111 (34.7)
Have conducted research after graduating from medical school	111 (34.7)
Have published any article in a journal	36 (11.3)

*IQR* Interquartile range

^a^Others: Military health clinic (*n* = 6), Private Polyclinic(*n* = 1), Private University(*n* = 1), Traditional & Complimentary Medicine Division(*n* = 1), University Health Clinic(*n* = 4)

### Validity

Flesch reading ease of the EBMQ was 61.2. Initially, we hypothesized that the “knowledge” domain would have two factors. However, EFA found that the “knowledge” domain had four factors: (“evidence-based medicine websites”, “evidence-based journals”, “type of studies” and “terms related to EBM”) after 9 items (item C1: “Clinical Practice Guidelines”, item C7: “Dynamed”, item C11: “InfoPoems”, item C4: “Cochrane”, item C8: “TRIP database”, item C15: “BestBETs”, item C9: “MEDLINE”, item C17: “Medscape” and item C16: “UpToDate”) were removed. This model explained 54.3% of the variation (Table 3).

**Table 3:** Exploratory factor analysis of the evidence-based medicine questionnaire

Original domains	After EFA was performed	Item No.	Item	Factor 1	Factor 2	Factor 3	KMO	AVE (%)	Bartlett’s test	CR
Knowledge	Evidence-based medicine websites (*n* = 6)	C6	Centre of Evidence-Based Medicine (CEBM)	0.605	–	–	0.834	43.0	< 0.001	0.662
		C10	ACP Journal Club	0.583	–	–				
		C5	Database of abstracts of reviews of effectiveness (DARE)	0.550	–	–				
		C13	InfoClinics	0.545	–	–				
		C2	Bandolier (published in Oxford)	0.495	–	–				
		C14	Centre of Reviews & Dissertation	0.477	–	–				
	Evidence-based journals (*n* = 2)	C12	BMJ Clinical Evidence	–	0.665	–	0.500	48.9	< 0.001	0.609
		C3	Evidence-Based Medicine (from BMJ publishing group)	–	0.658	–				
	Type of studies (*n* = 4)	K3	Case-control study	0.654	–	–	0.692	49.7	< 0.001	0.685
		K4	Randomized controlled trial	0.632	–	–				
		K1	Systematic review	0.622	–	–				
		K2	Meta-analysis	0.459	–	–				
	Terms related to EBM (*n* = 12)	K13	Publication bias	–	0.956	–	0.896	52.0	< 0.001	0.884
		K11	Confidence interval	–	0.817	–				
		K12	Heterogeneity	–	0.745	–				
		K16	Clinical effectiveness	–	0.642	–				
		K7	Odds ratio	–	0.607	–				
		K8	P-value	–	0.589	–				
		K15	Positive predictive value	–	0.569	–				
		K14	Test sensitivity and specificity	–	0.553	–				
		K10	Number needed to treat	–	0.531	–				
		K9	Level of evidence	–	0.524	–				
		K6	Absolute risk	–	0.436	–				
		K5	Relative risk	–	0.416	–				
Practice (*n* = 9)	Practice (*n* = 8)	P4	EBM improves my patient care	0.829	–	–	0.892	49.0	< 0.001	0.882
		P7	EBM guides my clinical decision making	0.817	–	–				
		P8	I prefer to manage patients based on EBM	0.759	–	–				
		P3	Reading research papers is important to me	0.739	–	–				
		P6	I can implement EBM in my clinical practice	0.727	–	–				
		P2	I trust the findings from research studies	0.662	–	–				
		P5	EBM reduces my workload	0.521	–	–				
		P1	I support EBM	0.456	–	–				
Barriers (*n* = 13)	Access (*n* = 6)	B4	I have time to practise EBM in my clinic	0.686		–	0.818	36.8	< 0.001	0.774
		B5	My clinic facilities are adequate to support the practice of EBM	0.675	–	–				
		B3	I have time to read research papers	0.633	–					
		B6	Research articles are easily available to me	0.632	–	–				
		B1	I am able to assess the quality of research.	0.543	–	–				
		B2	I have access to internet to practice EBM	0.435						
	Patient preferences (*n* = 2)	B8	My patients prefers me to practise EBM	–	0.754	–				
		B9	My patient believes in information that is based on evidence	–	0.754	–	0.500	56.8	< 0.001	0.725
	Support (*n* = 2)	B12	My colleagues support the practice of EBM	–	–	0.786				
		B13	My organization supports the practice of EBM	–	–	0.786	0.500	61.7	< 0.001	0.764

*EBM* Evidence-based medicine, *EFA* Exploratory Factor Analysis, *KMO* Keiser-Meyer-Olkin, *AVE* Average Variance Extracted, *CR* Composite Reliability

EFA found that the “practice” domain had only one factor with eight items after one item (item 9: “I prefer to manage patients based on my experience”) was removed. This model explained 49.0% of the variation (Table 3).

We hypothesized that the ‘barriers’ domain would only have one factor. However, EFA revealed that the ‘barriers’ domain has three factors (“access”, “support” and “patient’s preferences”) after three items were removed (item 7: “I can consult the specialist anytime to answer my queries”, item 10: “I have the authority to change the management of patients in my clinic” and item 11: “There are incentives for me to practice EBM”). This model explained 49.9% of the variation (Table 3).

#### Discriminative validity

In the “knowledge” domain, doctors who had EBM training had significant higher scores in 13 out of 24 items compared to those without training. In the “practice” domain, doctors who had EBM training had significant higher scores in 5 out 8 items compared to those without training. In the “barriers” domain, doctors who had EBM training had significant higher scores in 5 out of 10 items compared to those without training (Table 4).

**Table 4:** The discriminative validity of the Evidence-Based Medicine Questionnaire

Item	Details of item	With EBM training (*n* = 222) n(%)				Without EBM training (*n* = 98) n(%)				Chi-square	*p*-value
		Unaware	Aware but not used in clinical decision making	Have read it but not used in clinical decision making	Read and used in clinical decision making	Unaware	Aware but not used in clinical decision making	Have read it but not used in clinical decision making	Read and used in clinical decision making		
Knowledge Domain (Information sources related to EBM)											
C2	Bandolier	154(69.4)	35(15.8)	19(8.6)	14(6.3)	66(67.3)	13(13.3)	12(12.2)	7(7.1)	1.350	0.717
C5	DARE	155(18.9)	42(18.9)	16(7.2)	9(4.1)	71(72.4)	15(15.3)	12(12.2)	–	6.510	0.089
C6	CEBM	123(56.3)	69(31.1)	17(7.7)	11(5.0)	58(59.2)	22(22.4)	14(14.3)	4(4.1)	5.074	0.166
C10	ACP	147(66.2)	38(17.1)	24(10.8)	13(5.9)	63(64.3)	16(16.3)	14(14.3)	5(5.1)	0.825	0.844
C13	InfoClinics	152(68.5)	44(19.8)	18(8.1)	8(3.6)	63(64.3)	16(16.3)	10(10.2)	9(9.2)	4.946	0.176
C14	CRD	175(78.8)	31(14.0)	15(6.8)	1(0.5)	75(76.5)	12(12.2)	9(9.2)	2(2.0)	2.564	0.464
C3	EBM	10(4.5)	46(20.7)	66(29.7)	100(45.0)	4(4.1)	28(28.6)	24(24.5)	42(42.9)	2.577	0.462
C12	BMJ	26(11.7)	43(19.4)	73(32.9)	80(36.0)	10(10.2)	25(25.5)	22(22.4)	41(41.8)	4.442	0.218
Item	Details of item		With EBM training (*n* = 222) n(%)				Without EBM training (*n* = 98) n(%)			Chi-square	*p*-value
			Never heard and do not understand	Do not understand but would like to	Understand		Never heard and do not understand	Do not understand but would like to	Understand		
Knowledge Domain (Terms related to EBM)											
K1	Systematic review		6(2.7)	12(5.4)	204(91.9)		8(8.2)	8(8.2)	82(83.7)	5.975	0.050
K2	Meta-analysis		10(4.5)	14(6.3)	198(89.2)		6(6.1)	14(6.3)	198(89.2)	16.837	≤ 0.001*
K3	Case-control study		4(1.8)	8(3.6)	210(94.6)		5(5.1)	8(3.1)	90(91.8)	2.746	0.253
K4	Randomized controlled trial		4(1.8)	7(3.2)	211(95.0)		4(4.1)	1(1.0)	93(94.9)	2.651	0.266
K5	Relative risk		8(3.6)	25(11.3)	189(85.1)		9(9.2)	16(16.3)	73(74.5)	6.287	0.043*
K6	Absolute risk		8(3.6)	33(14.9)	181(81.5)		10(10.2)	15(15.3)	73(74.5)	5.699	0.058*
K7	Odds ratio		11(0.5)	60(27.0)	151(68.0)		14(14.3)	26(26.5)	58(59.2)	8.395	0.015*
K8	*P*-value		11(5.0)	38(17.1)	173(77.9)		14(14.3)	19(19.4)	65(66.3)	9.004	0.011*
K9	Level of evidence		7(3.2)	30(13.5)	185(83.3)		9(9.2)	19(19.4)	70(71.4)	7.686	0.021*
K10	Number needed to treat		11(5.0)	42(18.5)	170(76.6)		11(11.2)	20(20.4)	67(68.4)	4.640	0.098*
K11	Confidence interval		17(7.7)	61(27.5)	144(64.9)		21(21.4)	25(25.5)	52(53.1)	12.502	0.002*
K12	Heterogeneity		21(9.5)	74(33.3)	127(57.2)		22(22.4)	35(35.7)	41(41.8)	11.709	0.003*
K13	Publication bias		23(10.4)	65(29.3)	134(60.4)		23(23.5)	30(30.6)	45(45.9)	10.703	0.005*
K14	Test sensitivity and specificity		4(1.8)	25(11.3)	193(86.9)		11(11.2)	13(13.3)	74(75.5)	14.172	≤ 0.001*
K15	Positive predictive value		5(2.3)	36(16.2)	181(81.5)		5(2.3)	36(16.2)	181(81.5)	7.415	0.025*
K16	Clinical effectiveness		10(4.5)	48(21.6)	164(73.9)		16(16.3)	19(19.4)	63(64.3)	12.738	0.002*
Item	Details of item			With EBM training (*n* = 222) n(%)			Without EBM training (*n* = 98) n(%)			Chi-square	*p*-value
				Disagree	Neutral	Agree	Disagree	Neutral	Agree		
Practice Domain											
P1	I support EBM			1(0.5)	8(3.6)	213(95.9)	2(2.0)	6(6.1)	90(91.8)	2.941	0.230
P2	I trust the findings from research studies			1(0.5)	37(16.7)	184(82.9)	3(3.1)	13(13.3)	82(83.7)	4.216	0.121
P3	Reading research papers is important to me			–	25(11.3)	197(88.7)	4(4.1)	20(20.4)	74(75.5)	14.511	0.001*
P4	EBM improves my patient care			–	19(8.6)	203(91.4)	3(3.1)	8(8.2)	87(88.8)	6.862	0.032*
P5	EBM reduces my workload			22(9.9)	87(39.1)	113(50.9)	21(9.4)	39(17.5)	38(17.1)	8.838	0.012*
P6	I can implement EBM in my clinical practice			2(0.9)	23(10.3)	197(88.7)	3(1.3)	16(7.2)	79(35.5)	4.537	0.103
P7	EBM guides my clinical decision making			–	11(5.0)	211(95.0)	3(3.1)	9(9.2)	86(87.8)	9.130	0.010*
P8	I prefer to manage patients based on EBM			2(0.9)	36(16.2)	184(82.9)	3(3.1)	25(25.5)	70(71.4)	6.235	0.044*
Barriers Domain											
B1	I am able to assess the quality of research.			35(15.8)	76(34.2)	111(50.0)	17(17.3)	40(40.8)	41(41.8)	1.871	0.392
B2	I have access to internet to practice EBM			4(1.8)	18(8.1)	200(90.1)	11(11.2)	12(12.2)	75(76.5)	15.573	< 0.001*
B3	I have time to read research papers			25(11.3)	93(41.9)	104(46.8)	22(22.4)	39(39.8)	37(37.8)	7.142	0.028*
B4	I have time to practise EBM in my clinic			18(8.1)	60(27.0)	144(64.9)	144(64.9)	17(17.3)	32(32.7)	8.545	0.014*
B5	My clinic facilities are adequate to support the practice of EBM			47(20.2)	85(38.2)	90(40.5)	34(15.3)	34(15.3)	120(54.0)	6.935	0.031*
B6	Research articles are easily available to me			50(22.5)	71(32.0)	101(45.5)	40(40.8)	29(29.6)	29(29.6)	12.447	0.002*
B8	My patients prefers me to practise EBM			28(12.6)	138(62.2)	56(25.2)	15(15.3)	59(60.2)	24(24.5)	0.424	0.809
B9	My patient believes in information that is based on evidence			35(15.8)	95(42.8)	92(41.4)	11(11.2)	47(48.0)	40(40.8)	1.391	0.499
B12	My colleagues support the practice of EBM			13(5.9)	84(37.8)	125(56.3)	12(12.2)	36(36.7)	50(51.0)	3.922	1.141
B13	My organization supports the practice of EBM			12(5.4)	72(32.4)	138(62.2)	8(8.2)	33(33.7)	57(58.2)	1.038	0.595

*EBM* Evidence-based medicine

*p ≤ 0.05 is significant

#### Reliability

Cronbach alpha for the overall EBMQ was 0.909, whilst individual domains ranged from 0.657 to 0.933 (Table 4). All corrected item-total correlation (CITC) values were > 0.2. At retest, 185 participants completed the EBMQ (response rate = 57.85%), as *n* = 23 (42%) were uncontactable. Thirty items had good and fair correlations (*r* = 0.418–0.620) while 12 items had low correlations (r = < 0.4). (Table 5).

**Table 5:** The psychometric properties of the Evidence-Based Medicine Questionnaire

No.	Items	Test-Retest Reliability						
		Corrected Item-total Correlation	Cronbach’s alpha if items is deleted	Test (*n* = 320)		Retest (*n* = 184)		ICC
				Mean (SD)	Median	Mean (SD)	Median	
Knowledge Domain								
C2	Bandolier (Published in Oxford)	0.487	0.811	1.31 (0.650)	1.00	1.68 (1.003)	1.00	0.567
C5	Database of abstracts of reviews of effectiveness(DARE)	0.630	0.778	1.54 (0.916)	1.00	1.67 (0.922)	1.00	0.485
C6	Centre of Evidence-Based Medicine (CEBM)	0.630	0.777	1.44 (0.769)	1.00	1.76 (0.937)	1.00	0.453
C10	ACP Journal Club	0.570	0.791	1.62 (0.844)	1.00	1.55 (0.886)	1.00	0.333
C13	InfoClinics	0.566	0.791	1.58 (0.907)	1.00	1.63 (0.913)	1.00	0.418
C14	Centre of Reviews & Dissertation (CRD)	0.650	0.780	1.52 (0.863)	1.00	1.48 (0.815)	1.00	0.396
C3	Evidence-based medicine (EBM)	0.492	–	1.52 (0.863)	3.00	3.21 (0.881)	3.00	0.416
C12	BMJ Clinical Evidence	0.492	–	3.23 (0.868)	3.00	2.90 (0.997)	3.00	0.379
K1	Systematic review	0.774	0.866	4.19 (0.775)	4.00	4.23(0.814)	4.00	0.421
K2	Meta-analysis	0.718	0.887	2.79(0.516)	3.00	4.10(0.793)	4.00	0.463
K3	Case-control study	0.826	0.848	2.91(0.373)	3.00	4.28(0.681)	4.00	0.497
K4	Randomized controlled trial	0.777	0.866	2.93(0.346)	3.00	4.37(0.686)	4.00	0.522
K5	Relative risk	0.747	0.927	2.77(0.535)	3.00	4.04(0.741)	3.00	0.450
K6	Absolute risk	0.763	0.926	2.74(0.554)	3.00	4.01(0.775)	4.00	0.561
K7	Odds ratio	0.742	0.926	2.58(0.634)	3.00	3.82(0.822)	4.00	0.506
K8	P-value	0.713	0.927	2.67(0.616)	3.00	4.00(0.803)	4.00	0.487
K9	Level of evidence	0.721	0.927	2.75(0.538)	3.00	4.06(0.846)	4.00	0.359
K10	Number needed to treat	0.676	0.929	2.67(0.599)	3.00	3.95(0.943)	4.00	0.528
K11	Confidence interval	0.757	0.926	2.49(0.699)	3.00	3.78(0.882)	4.00	0.529
K12	Heterogeneity	0.663	0.930	2.39(0.713)	3.00	3.54(0.950)	4.00	0.483
K13	Publication bias	0.686	0.929	2.42(0.729)	3.00	3.58(0.997)	4.00	0.580
K14	Test sensitivity and specificity	0.697	0.928	2.79(0.512)	3.00	4.24(0.734)	4.00	0.504
K15	Positive predictive value	0.707	0.928	2.74(0.522)	3.00	4.06(0.861)	4.00	0.503
K16	Clinical effectiveness	0.667	0.929	2.63(0.630)	3.00	2.89(0.938)	4.00	0.570
Practice Domain								
P1	I support EBM	0.417	0.875	2.94 (0.279)	3.00	4.43 (0.648)	4.00	0.605
P2	I trust the findings from research studies	0.618	0.854	4.02 (0.683)	4.00	4.09 (0.611)	4.00	0.323
P3	Reading research papers is important to me	0.684	0.846	4.06 (0.687)	4.00	4.06 (0.679)	4.00	0.477
P4	EBM improves my patient care	0.765	0.838	4.18 (0.642)	4.00	4.27 (0.626)	4.00	0.301
P5	EBM reduces my workload	0.499	0.877	3.44 (0.898)	3.00	3.43 (0.830)	3.00	0.532
P6	I can implement EBM in my clinical practice	0.682	0.846	4.04 (0.661)	4.00	3.90 (0.743)	4.00	0.532
P7	EBM guides my clinical decision making	0.748	0.841	4.18 (0.607)	4.00	4.10 (0.600)	4.00	0.344
P8	I prefer to manage patients based on EBM	0.699	0.844	4.01 (0.713)	4.00	4.02 (0.689)	4.00	0.422
Barrier Domain								
B1	I am able to assess the quality of research.	0.472	0.747	2.31 (0.736)	2.00	3.34 (0.808)	3.00	0.475
B2	I have access to internet to practice EBM	0.386	0.767	2.81 (0.497)	3.00	3.83 (0.874)	4.00	0.388
B3	I have time to read research papers	0.546	0.728	3.32 (0.803)	3.00	3.29 (0.795)	3.00	0.494
B4	I have time to practise EBM in my clinic	0.583	0.718	3.55 (0.810)	4.00	3.45 (0.774)	4.00	0.356
B5	My clinic facilities are adequate to support the practice of EBM	0.583	0.718	3.13 (0.894)	3.00	3.30 (2.367)	3.00	0.142
B6	Research articles are easily available to me	0.547	0.731	3.16 (0.982)	3.00	3.06 (0.942)	3.00	0.275
B8	My patients prefers me to practise EBM	0.569	–	3.14 (0.798)	3.00	3.24 (0.690)	3.00	0.323
B9	My patient believes in information that is based on evidence	0.569	–	3.29 (0.853)	3.00	3.41 (0.717)	3.00	0.547
B12	My colleagues support the practice of EBM	0.618	–	3.53 (0.795)	4.00	3.53 (0.752)	4.00	0.620
B13	My organization supports the practice of EBM	0.618	–	3.63 (0.756)	4.00	3.53 (0.771)	4.00	0.471

*ICC* Intraclass correlation

*Statistically significant at *p* < 0.05

## Discussion

The EBMQ was found to be a valid and reliable instrument to assess the knowledge, practice and barriers of primary care physicians regarding the implementation of EBM. The final EBMQ consists of 42 items with 8 domains after 13 items were removed. The Flesch reading ease was 61.2. This indicates that the EBMQ can be easily understood by 13–15 years old students who study English as a first language [23].

Initially, we hypothesized that there were two factors in the “knowledge” domain: “sources related to EBM” and “terms related to EBM”. However, EFA revealed that the EBMQ had four factors: “evidence-based medicine websites”, “evidence-based journals”, “terms related to EBM” and “type of studies” after 9 items were removed. This was because “sources related to EBM” was further divided into another three factors. It is not surprising because knowledge is a broad concept that can be further recategorized. EFA revealed that the “practice” domain had one factor which concurred with our initial hypothesis. One item (item P9: “I prefer to manage patients based on my experience”) was removed as this was regarding doctors’experience rather than their practice. Initially, we hypothesized that there was one factor in the “barriers” domain. However, EFA revealed that there were three factors: ‘access to resources’, ‘patient preferences towards EBM’ and ‘support from the management’ after three items were removed. This may be because instead of one barrier, EFA had re-grouped into three factors that provided a better description of barriers encountered by the primary care physicians. As highlighted in literature [9, 31], there are many barriers to practice EBM and some of it were also categorized according the specific and types of barriers.

The EBMQ was able to discriminate the knowledge, practice and barriers between doctors with and without EBM training. In the knowledge domain, there were significant differences for all items in the “terms related to EBM”. This is not surprising as doctors with EBM training would have been exposed to these terms. No differences was found between those with and without EBM training in “information sources related to EBM” as those who did not attend EBM training could still access online information resources. Several studies were found to improve knowledge but did not report in detail which areas on knowledge. Hence, we could not compare their findings to our studies [32–35].

Our findings also showed that doctors with EBM training had better practice of EBM. This differed from several studies which reported changes in practice [32, 36–39] and some reported no changes in practice [35, 40]. However, the authors commented that these findings were not meaningful as it was self-perceived. Other than that, in our findings, doctors who attended EBM training had less barriers regarding the implementation of EBM in their clinical practice. They seemed to have better access to resources, more patients had a positive attitude towards EBM, and better support from management to practice EBM compared to those without EBM training. This could be because doctors with EBM training knew how to overcome problems that would prevent them from practicing EBM. In the systematic review [41], the barriers in the implementation of EBM remains unclear as it was not reported.

The overall Cronbach’s alpha as well as the individual domains were > 0.7. This indicates that the EBMQ has adequate psychometric properties, which was similar to previous studies [12, 14–16, 19, 42]. The majority (71.4%) of the items in EBMQ had good and fair correlation at test-retest, which indicates that the EBMQ has achieved adequate reliability. The reliability testing two weeks later did not affect the methodology as the acceptable time interval for test-retest reliability is approximately 2 weeks [28]. The discriminative validity was performed using the baseline data and not after retest which then impact on the methodology.

To our knowledge, this was the first validation study assessed the discriminative validity (i.e. between doctors with and without EBM training) that assessed their implementation of EBM. One of the limitations of this study was that participants were recruited whilst attending a Family Medicine module workshop. This may mean that participants that were recruited may be more interested in the practice of EBM as they are already interested in furthering their postgraduate studies. This cohort are likely to be more interested with the practice of EBM as they are more incline to further their studies rather than the normal general practitioners. Hence, our result may not be generalizable.

## Conclusions

The EBMQ was found to be a valid and reliable instrument to assess the knowledge, practice and barriers of primary care physicians towards EBM in Malaysia. The EBMQ can be used to assess doctors’ practices and barriers in the implementation of EBM. Information gathered from the administration of the EBMQ will assist policy makers to identify the level of knowledge, practice and barriers of EBM and to improve its uptake in clinical practice. Although the findings of this study are not generalizable, they may be of interest to primary care physicians in other countries.

## Additional file

Additional file 1: The Evidence-Based Medicine Questionnaire (EBMQ), The final version of EBMQ to assess doctors’ knowledge, practice and barriers regarding the implementation of Evidence-Based Medicine in primary care. (DOCX 228 kb)

## Abbreviations

AVE: Average variance extracted
CITC: Corrected item-total correlation
CR: Composite reliability
EBM: Evidence-based medicine
EBMQ: Evidence-based medicine questionnaire
EFA: Exploratory factor analysis
ICC: Intra-correlation coefficient
IQR: Interquartile range
KMO: Kaiser-Meyer-Oklin
SPSS: Statistical Package for Social Sciences
