# Supplementary material for: Development and validation of the Evidence Based Medicine Questionnaire (EBMQ) to assess doctors’ knowledge, practice and barriers regarding the implementation of evidence-based medicine in primary care
Source: BMC Fam Pract. 2018 Jun 23;19:98. doi: 10.1186/s12875-018-0779-5 (PMC6015654; doi:10.1186/s12875-018-0779-5)
Supplement: Supplementary file 1 — The Evidence-Based Medicine Questionnaire (EBMQ), The final version of EBMQ to assess doctors’ knowledge, practice and barriers regarding the implementation of Evidence-Based Medicine in primary care. (DOCX 228 kb) [file 12875_2018_779_MOESM1_ESM.docx]

## **EVIDENCE-BASED MEDICINE QUESTIONNAIRE (EBMQ)**

# SECTion A: DemogRaphic Profile

We require you to provide your particulars as we need you to answer this survey again in **2 weeks’ time**. All the information you provide will be kept confidential.

Name: ………………………………………………………………..

E-mail address: ………………………………………………………

Phone no: …………………………………………………………….

1. Date of birth: _ _ / _ _ / _ _ (dd/ mm/ yy)

2. Your gender: Male Female

3. What is your current position? (Please tick only **ONE** answer)

Medical officer

General practitioner

Family Medicine trainee (Currently undergoing the MMed programme)

Family Medicine Specialist in the government sector

Family Medicine Specialist in the private sector

Academic lecturer in a public university

Academic lecturer in a private university

Others. Please specify: .................................................................................

4. Where is your current place of work?

Government hospital

Private hospital

University hospital

Government health clinic (Kesihatan Kesihatan)

Private clinic

Others. Please specify: ................................................................

5. Year of graduation from medical school: ..............................

6. Do you have any postgraduate qualification?

Yes PhD

Masters

Diploma

Others. Please specify: ………………………………………………..

No

# Section B: Information sources

7. On average, how often do you look for clinical information from medical literature?

*(This can be from textbooks, academic journals or online databases)*

Always (several times a week)

Often (once a week)

Sometimes (at least once a month)

Rarely (once in a few months)

Never in the past 1 year

8. **In the past 1 year**, how often did you look for medical information from the following sources?

For each item, please tick **ONE** answer that best represents your view.

| Sources of information | **Always**  ***(several times a week)*** | **Often**  ***(once a week)*** | **Sometimes**  ***(at least once a month)*** | **Rarely**  ***(once in a few months)*** | **Never in the past 1 year** | **Not available** |
| --- | --- | --- | --- | --- | --- | --- |
| 1. Textbooks |  |  |  |  |  |  |
| 2. Journal articles |  |  |  |  |  |  |
| 3. Clinical Practice Guidelines (CPG) |  |  |  |  |  |  |
| 4. Online database  (e.g.: MEDLINE, Cochrane and TRIP database) |  |  |  |  |  |  |
| 5. Medical websites  (e.g.: e-medicine, UptoDate, Medscape) |  |  |  |  |  |  |
| 6. General database  (e.g. Google, Wikipedia) |  |  |  |  |  |  |
| 7. Social Media  (e.g.: WhatsApp  WeChat, Facebook) |  |  |  |  |  |  |
| 8. Medical apps  (e.g.: ePocrates, Medical Calculator) |  |  |  |  |  |  |
| 9. Peers/ Colleagues |  |  |  |  |  |  |
| 10. Family Medicine Specialist |  |  |  |  |  |  |
| 11. Hospital Specialist |  |  |  |  |  |  |
| 12. Pharmaceutical representatives |  |  |  |  |  |  |
| 13. Conferences/ Talks/Seminars/ Forum |  |  |  |  |  |  |

9. Have you heard of the term “evidence-based medicine” (EBM)?

Yes No

10. Have you ever attended a course or workshop on EBM?

Yes No

11. Have you ever received any formal training in the following area?

- Question formulation Yes No
- Literature search Yes No
- Critical appraisal Yes No

1. Did you conduct any research after graduating from medical school?

*(As an investigator or involved in data collection)*

Yes No

1. Have you published any article in a journal?

Yes. Please state how many?  …………..

No

# SECTION C: **KNOWLEDGE** AND PRACTICE IN IMPLEMENTING EBM

1. Here are some of the resources available and used in practising EBM. Please indicate those which you are aware of and have used in clinical decision making.

For each item, please tick **ONE** answer that best represents your view.

|  | **Sources of information** | **Unaware** | **Aware but not used in clinical decision making** | **Have read it but not used in clinical decision making** | **Read and used in clinical decision making** |
| --- | --- | --- | --- | --- | --- |
| 1. | Bandolier (Published in Oxford) |  |  |  |  |
| 2. | Evidence Based Medicine (from BMJ publishing group) |  |  |  |  |
| 3. | Database of abstracts of reviews of effectiveness (DARE) |  |  |  |  |
| 4. | Centre of Evidence-based medicine (CEBM) |  |  |  |  |
| 5. | ACP Journal Club |  |  |  |  |
| 6. | BMJ Clinical Evidence |  |  |  |  |
| 7. | InfoClinics |  |  |  |  |
| 8. | Centre of Reviews & Dissertation |  |  |  |  |

15. Please state any other online resources you have used in clinical decision making:

1. …………………………………..…
2. …………………………….……….
3. ……………………………………..

16. Below are terms commonly used in EBM.

For each item, please tick **ONE** answer that best represents your view.

|  | **Terms** | **Never heard of this term before** | **Heard of this term but do not understand what it means** | **Do not understand this term but would like to** | **Have some understanding of this term** | **Understand this term well and able to explain what it means to others** |
| --- | --- | --- | --- | --- | --- | --- |
| 1. | Systematic review |  |  |  |  |  |
| 2. | Meta-analysis |  |  |  |  |  |
| 3. | Case-control study |  |  |  |  |  |
| 4. | Randomized controlled trial |  |  |  |  |  |
| 5. | Relative risk |  |  |  |  |  |
| 6. | Absolute risk |  |  |  |  |  |
| 7. | Odds ratio |  |  |  |  |  |
| 8. | P-value |  |  |  |  |  |
| 9. | Level of evidence |  |  |  |  |  |
| 10. | Number needed to treat |  |  |  |  |  |
| 11. | Confidence interval |  |  |  |  |  |
| 12. | Heterogeneity |  |  |  |  |  |
| 13. | Publication bias |  |  |  |  |  |
| 14. | Test sensitivity and specificity |  |  |  |  |  |
| 15. | Positive predictive value |  |  |  |  |  |
| 16. | Clinical effectiveness |  |  |  |  |  |

17. What is your opinion regarding EBM?

For each item, please tick **ONE** answer that best represents your view.

|  | **Strongly disagree** | **Disagree** | **Neither agree nor Disagree** | **Agree** | **Strongly agree** |
| --- | --- | --- | --- | --- | --- |
| 1. I support EBM |  |  |  |  |  |
| 2. I trust the findings from research studies |  |  |  |  |  |
| 3. Reading research papers is important to me |  |  |  |  |  |
| 4. EBM improves my patient care |  |  |  |  |  |
| 5. EBM reduces my workload |  |  |  |  |  |
| 6. I can implement EBM in my clinical practice |  |  |  |  |  |
| 7. EBM guides my clinical decision making |  |  |  |  |  |
| 8. I prefer to manage patients based on EBM |  |  |  |  |  |

# Section D: Barriers and facilitators to evidence-based practice

|  | | | **Strongly Disagree** | | **Disagree** | | **Neither agree nor disagree** | | **Agree** | | **Strongly agree** | |
| --- | --- | --- | --- | --- | --- | --- | --- | --- | --- | --- | --- | --- |
| 1. | I am able to assess the quality of research. |  | |  | |  | |  | |  | |  |
| 2. | I have access to internet to practice EBM |  | |  | |  | |  | |  | |  |
| 3. | I have time to read research papers. |  | |  | |  | |  | |  | |  |
| 4. | I have time to practise EBM in my clinic |  | |  | |  | |  | |  | |  |
| 5. | My clinic facilities are adequate to support the practice of EBM. |  | |  | |  | |  | |  | |  |
| 6. | Research articles are easily available to me. |  | |  | |  | |  | |  | |  |
| 7. | My patient prefers me to practise EBM |  | |  | |  | |  | |  | |  |
| 8. | My patient believes in information that is based on evidence |  | |  | |  | |  | |  | |  |
| 9. | My colleagues support the practice of EBM. |  | |  | |  | |  | |  | |  |
| 10. | My organization supports the practice of EBM. |  | |  | |  | |  | |  | |  |

18. Here are some difficulties you may face when practising EBM.

For each item, please tick **ONE** answer that best represents your view.

19. Please suggest ways to improve the practice of EBM in your clinical practice.

………………………………………………………………………………………………………………………………………………………………………………………………………………………………………………………………………………………………………………………………………………………………………

........................................................................................................................................................................................................................................................................................................................................................................

# SECTION D: Needs to practice EBM

20. If there is a service which provides evidence-based answers to your clinical queries, would you be interested to use it?

Yes

No

21. If we were to provide this service, how would you prefer it to be delivered? Please tick only **ONE** answer.

Online messaging service. E.g.: WhatsApp

Social media. E.g.: Facebook

Mobile apps. E.g.: Epocrates

Telephone helpline

Website with archive of answers. E.g.: TRIP database

Personal email

Online forum

Others. Please specify: ………………………………………………………………..

22. If we were to provide this service, what would be a reasonable time for us to deliver the answer to you?

………….. days

For further enquiry, please contact:

Ranita Hisham,

Department of Primary Care Medicine,

Faculty of Medicine,

University of Malaya.

Corresponding email: [ranita@um.edu.my](mailto:ranita@um.edu.my)

*Thank you*
